# Risk factors for post-traumatic osteoarthritis following surgical treatment of acetabular posterior wall fractures: a retrospective study

**DOI:** 10.1038/s41598-026-41893-6

**Published:** 2026-02-26

**Authors:** Gongwu Yuan, Xi Ke, Junhong Lian, Guodong Wang, Sheng-Hui Lan, Xi-Ming Liu

**Affiliations:** 1 Department of Orthopedic Surgery, Hubei Provincial Hospital of Integrated Chinese and Western Medicine, Wuhan, Hubei China; 2 Department of Nursing, Hubei Provincial Hospital of Integrated Chinese and Western Medicine, Wuhan, Hubei China; 3 Department of Orthopedics, General Hospital of Central Theater Command, Wuhan, Hubei China; 4 College of Acupuncture and Orthopedics, Hubei University of Chinese Medicine, Wuhan, Hubei China; 5 Department of Orthopedics, The Eighth People’s Hospital, Jiangsu University, Shanghai, China; 6 Department of Orthopedics, Xuhui Branch of The Sixth People’s Hospital, Shanghai Jiao Tong University, Shanghai, China

**Keywords:** Acetabular fracture, Posterior wall fracture, Post-traumatic osteoarthritis, Risk factors, Postoperative complications, Diseases, Health care, Medical research, Risk factors

## Abstract

Posterior wall fractures, commonly caused by high-energy mechanisms like motor vehicle collisions or falls, are frequently associated with hip dislocation and complex intra-articular injuries. Despite surgical advances, post-traumatic osteoarthritis (PTOA) remains a prevalent complication, often leading to significant functional impairment and eventual joint replacement. Identifying potentially modifiable risk factors may help improve long-term outcomes. This study aimed to identify independent risk factors associated with the development of post-traumatic osteoarthritis (PTOA) following surgical treatment of posterior wall acetabular fractures. A multicenter retrospective cohort study was conducted on 159 patients with isolated posterior wall acetabular fractures treated with open reduction and internal fixation between January 2017 and June 2023. All patients had a minimum of 24 months of clinical and radiographic follow-up. Univariate and multivariate logistic regression were used to analyze potential risk factors. The overall incidence of PTOA was 23.9%, with a median onset of 18 months after surgery. Multivariate logistic regression analysis identified fracture comminution, joint surface impaction, femoral head necrosis, non-anatomical reduction, posterior wall defect ≥ 50%, and delayed surgical intervention (> 2 weeks) as factors independently associated with an increased risk of PTOA (all *p* < 0.05). In addition, longer surgical duration was associated with a higher likelihood of PTOA. Multiple factors were independently associated with the development of post-traumatic osteoarthritis following posterior wall acetabular fractures, including fracture comminution, articular surface impaction, reduction quality, femoral head necrosis, posterior wall defect, and delayed surgical intervention. Anatomical reduction and earlier surgery were associated with a lower risk of PTOA in this cohort. These findings may assist in risk stratification and perioperative decision-making.

## Introduction

 Posterior wall fractures account for 14%–25% of acetabular fractures, often resulting from high-energy trauma such as motor vehicle collisions or falls from height, and are frequently associated with posterior hip dislocation, complicating treatment^1,2^. Achieving anatomical reduction and stable fixation is challenging, particularly in cases involving comminution or articular surface impaction^3^. Even with adequate postoperative reduction, patients may still experience functional impairment, suggesting that other factors may be associated with long-term outcomes^4^. Additionally, the incidence of acetabular fractures in the elderly, due to low-energy falls and osteoporosis, is rising, further complicating surgical management^5^.

Common complications of posterior wall fracture fixation include sciatic nerve injury, femoral head osteonecrosis, heterotopic ossification, and post-traumatic osteoarthritis (PTOA), which is one of the most frequent and debilitating long-term outcomes. Post-traumatic osteoarthritis occurs in 29.4% of transverse acetabular fractures within two years, with even higher rates in fractures involving the posterior wall^6^. Risk factors for PTOA include comminution, cartilage damage, poor reduction, and residual joint instability^7^. Even with anatomical reduction, initial cartilage damage and subtle postoperative instability may lead to PTOA, which can result in significant joint dysfunction and ultimately require total hip arthroplasty^7,8^.

While several studies have examined the risk factors for PTOA after acetabular fractures in general, specific evidence for posterior wall fractures remains limited. This study aims to identify independent risk factors for PTOA following posterior wall fractures, which may guide personalized surgical planning and help reduce PTOA incidence, improving long-term outcomes.

## Materials and methods

### Study design and population

This study was approved by the Medical Ethics Committee of Hubei Provincial Hospital of Integrated Chinese & Western Medicine. All methods were carried out in accordance with relevant guidelines and regulations, including the principles of the Declaration of Helsinki. Prior to their inclusion in the study, written informed consent was obtained from all participating patients. This multicenter retrospective cohort study included patients diagnosed with isolated posterior wall acetabular fractures who underwent surgical treatment between January 2017 and June 2023 at two tertiary trauma centers: Hubei Hospital of Integrated Traditional Chinese and Western Medicine and the General Hospital of the Central Theater Command of the PLA. The clinical data used in this retrospective study were accessed for research purposes between June 1, 2024, and December 31, 2024.To ensure patient confidentiality and comply with ethical standards, all patient identifiers (e.g., names, identification numbers, contact information) were anonymized and de-identified prior to analysis. The authors did not have access to any information that could identify individual participants during or after data collection. Inclusion criteria were: (1) diagnosis confirmed by CT as a unilateral posterior wall fracture based on Letournel classification^9^; (2) treatment with open reduction and internal fixation (ORIF); (3) age ≥ 18 years; and (4) a minimum follow-up of 24 months with complete radiological and clinical records. Patients were excluded if they had concurrent pelvic ring injuries (e.g., sacral fractures or pubic symphysis separation), pre-existing hip diseases (e.g., osteoarthritis or avascular necrosis), pathological fractures, or incomplete follow-up data.

### Surgical technique and postoperative protocol

All surgeries were performed by senior orthopedic trauma surgeons using the Kocher-Langenbeck posterior approach^10^. Internal fixation was achieved with reconstruction plates and screws according to standard protocols. A unified rehabilitation strategy was employed: non-weight-bearing mobilization during the first 6 weeks, partial weight-bearing (≤ 20 kg) between weeks 6 and 12, and full weight-bearing permitted after 12 weeks based on radiographic signs of fracture healing. Postoperative reduction quality was graded according to the Matta criteria based on computed tomography (CT) or plain radiographs: anatomical (≤ 1 mm step-off), imperfect (2–3 mm), or poor (> 3 mm)^11^. Patients were followed regularly at 3 months, 6 months, 12 months, and annually thereafter for clinical and radiological assessment.

### Patient-reported outcome measures

**Fig. 1:**
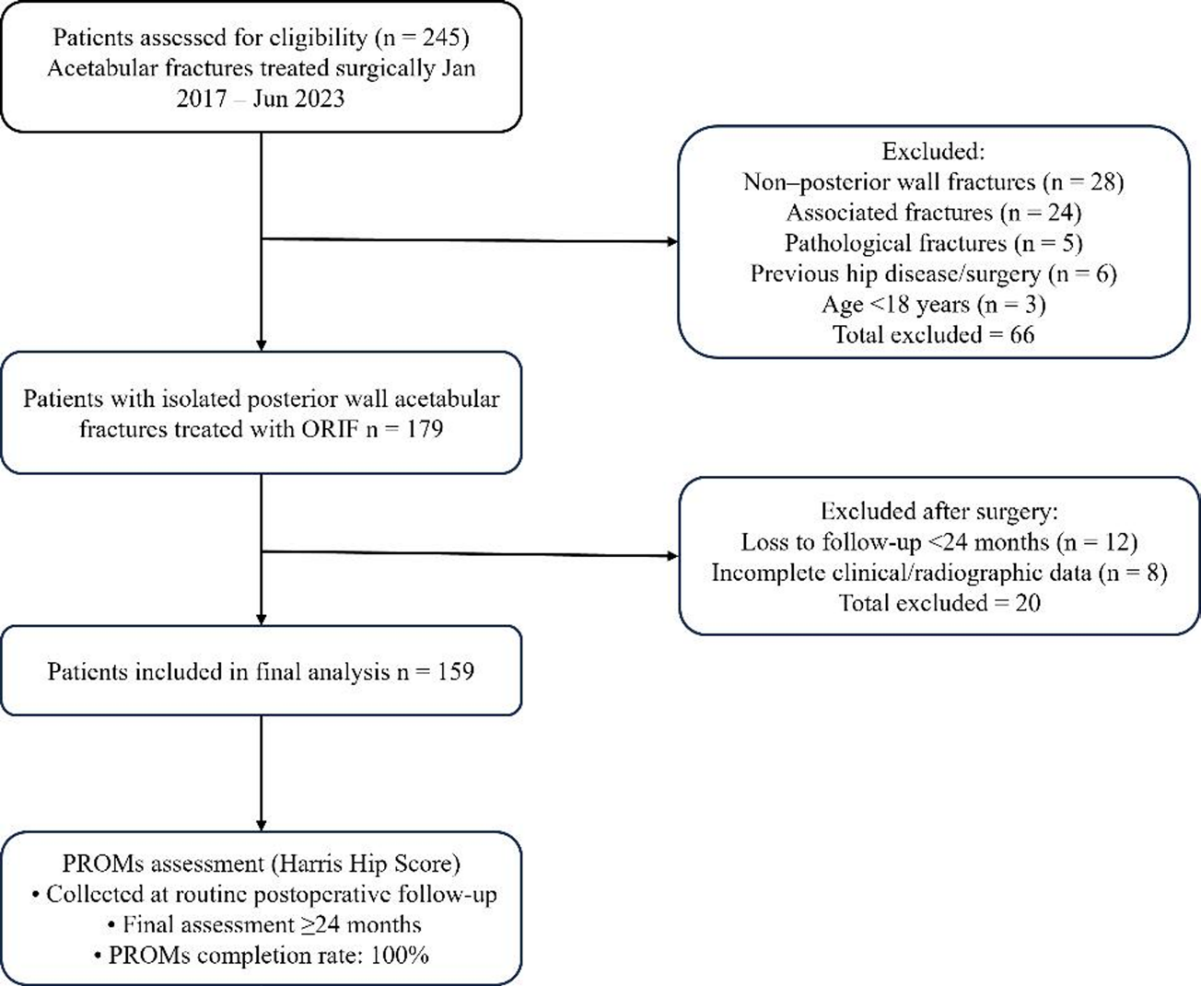
STROBE flow diagram of patient selection and study cohort formation. STROBE flow diagram illustrating patient selection, exclusion criteria, and inclusion in the final analysis. PROMs, patient-reported outcome measures; HHS, Harris Hip Score.

Patient-reported functional outcomes were assessed using the Harris Hip Score (HHS). The HHS was collected at routine postoperative follow-up visits, with the final PROMs assessment recorded at the most recent follow-up visit (minimum follow-up of 24 months). Importantly, completion of the HHS was required for inclusion in the final analysis. All patients included in the study completed the HHS assessment at final follow-up, resulting in a PROMs follow-up rate of 100% (Fig. 1).

### Data collection and variables

Clinical data were collected across four primary domains: patient demographics, injury characteristics, treatment-related factors, and postoperative outcomes. Demographic variables included age, sex, body mass index (BMI), bone mineral density (assessed by dual-energy X-ray absorptiometry, DEXA), and the presence of osteoporosis (defined as a T-score ≤ − 2.5 at the femoral neck or lumbar spine). Additional factors such as smoking status, alcohol use, and comorbidities (e.g., hypertension, diabetes) were also recorded. Injury-related variables included fracture comminution, displacement magnitude, articular surface impaction, hip dislocation, mechanism of injury, and associated femoral head injuries (such as cartilage damage or fracture). For clarity, joint surface impaction in this study refers exclusively to compression of the acetabular articular surface, whereas femoral head cartilage injury describes cartilage damage on the femoral head side. These two conditions involve different anatomical locations and were evaluated as separate entities. The extent of the posterior wall defect was measured on three-dimensional CT reconstructions and categorized as < 33%, 33–50%, or > 50% of the posterior acetabular arc^12^.Treatment-related variables encompassed time from injury to surgery (≤ 2 weeks or > 2 weeks), fixation method (screws, plates, or combined), fracture reduction quality (anatomical, imperfect, or poor), operative time, and intraoperative blood loss. Femoral head cartilage injury was assessed intraoperatively by direct visualization after joint exposure. Cartilage injury was defined as any macroscopic cartilage abnormality, including surface fissuring, partial-thickness defects, or full-thickness cartilage loss. For the purpose of analysis, cartilage injury was recorded as a binary variable (presence or absence). Preoperative imaging was not used to determine cartilage status. Operative duration and estimated blood loss were obtained from standardized anesthesia and surgical records. Postoperative outcomes included complications such as avascular necrosis of the femoral head (graded by the Ficat classification^13^ and heterotopic ossification (graded by the Brooker classification^14^. Functional outcomes were evaluated using the HHS^15^, with scores < 70 considered indicative of poor functional recovery. Harris Hip Score assessments were conducted by trained orthopedic clinicians at each follow-up interval.

The selection of candidate variables for multivariable analysis was guided by prior literature and clinical relevance. Fracture-related characteristics such as comminution, articular impaction, and posterior wall involvement have been shown to influence long-term outcomes following acetabular fracture fixation, particularly in posterior wall fractures^7,9^. Surgical quality indicators, especially reduction quality as defined by the Matta criteria, are well-established determinants of long-term joint survivorship and post-traumatic osteoarthritis^11,16^. In addition, postoperative joint integrity, including femoral head necrosis and traumatic cartilage damage, has been consistently associated with inferior outcomes despite anatomical reduction^8,17^. Variables such as time to surgery and operative duration were included based on both prior evidence and clinical plausibility, as these factors may affect soft-tissue conditions, reduction quality, and joint congruency.

### Diagnostic criteria for PTOA

The diagnosis of PTOA was based on a combination of clinical symptoms and radiographic findings. Clinical criteria included hip pain associated with activity and a HHS below 70 points. Radiographic criteria included joint space narrowing > 50%, presence of osteophytes, and a Kellgren-Lawrence grade ≥ 2^18^. All assessments were independently performed by two experienced radiologists or orthopedic surgeons. In cases of disagreement, a consensus was reached through joint review. Harris Hip Score was used to assess postoperative hip function. An HHS < 70 was considered indicative of poor clinical outcome as part of the composite definition of post-traumatic osteoarthritis. The HHS was otherwise analyzed descriptively and was not included as an independent predictor in regression analyses.

### Statistical analysis

Statistical analyses were performed using SPSS version 26.0 (IBM Corp., Armonk, NY). Descriptive statistics were used to summarize demographic and clinical data. Continuous variables were presented as mean ± standard deviation or median (IQR), and categorical variables as frequencies and percentages. Univariate analyses were conducted to identify potential predictors of PTOA using chi-square or Fisher’s exact test for categorical variables, and independent t-tests or Mann-Whitney U tests for continuous variables, as appropriate. Variables with a p-value < 0.10 in univariate analysis were initially considered for inclusion in the multivariable logistic regression model. However, to avoid model overfitting and multicollinearity, variables demonstrating substantial clinical overlap or strong intercorrelation were further evaluated. When closely related variables reflected similar clinical constructs (for example, fracture severity or surgical complexity), only the most clinically relevant or representative variable was retained for multivariable analysis. Reduction quality was categorized as anatomical, imperfect, and poor, with anatomical reduction as the reference category. Posterior wall defect size was categorized as < 33%, 33–50%, and > 50%, with < 33% used as the reference category. Multicollinearity among candidate variables was assessed using variance inflation factors (VIFs). All included variables demonstrated VIF values < 5, indicating no significant multicollinearity. Results were reported as odds ratios (OR) with 95% confidence intervals (CI). A two-sided p-value < 0.05 was considered statistically significant.

## Results

### Follow-up outcomes

A total of 159 patients with posterior wall acetabular fractures were included in the study, all of whom were followed up for an average of 38.4 ± 10.2 months (range, 24–60 months) postoperatively. During the follow-up period, the overall incidence of PTOA was 23.9% (38/159), with a median time to diagnosis of 18 months post-surgery (interquartile range: 12–28 months). Among the 38 patients who developed PTOA, 12 (31.6%) underwent total hip replacement (THA) within 24 months postoperatively. Complete PROMs data were available for all patients included in the final analysis. Complete PROMs data (HHS) at final follow-up were available for all patients included in the final analysis. Missing data were minimal, and all variables included in the analyses were complete. Therefore, a complete-case analysis was performed.

### Baseline characteristics

As shown in Table 1, no statistically significant differences were observed in most baseline characteristics between patients who developed PTOA and those who did not (all *p* > 0.05). The two groups were comparable with respect to age, sex distribution, BMI, fracture type and displacement, postoperative weight-bearing time, and the overall baseline distribution of reduction quality. Variables including time to surgery, severity of articular damage, and intraoperative blood loss also did not differ significantly between groups. In contrast, the incidence of postoperative complications was significantly higher in the PTOA group than in the non-PTOA group (31.6% vs. 5.0%, *p* = 0.001).

**Table 1:** Baseline characteristics of patients.

Variable	PTOA group (*n* = 38)	Non-PTOA group (*n* = 121)	*p*-value
Age (years)	47.2 ± 9.8	45.6 ± 10.2	0.412
Sex (Male)	24 (63.2%)	73 (60.3%)	0.788
BMI (kg/m²)	25.2 ± 3.0	24.6 ± 3.1	0.135
Fracture type (Comminuted)	15 (39.5%)	45 (37.2%)	0.795
Fracture displacement ≥ 2 mm	12 (31.6%)	28 (23.1%)	0.236
Anatomical reduction	30 (78.9%)	87 (71.9%)	0.495
Postoperative weight-bearing < 6 weeks	22 (57.9%)	55 (45.5%)	0.287
Intraoperative blood loss (mL)	485.3 ± 100.2	460.1 ± 120.3	0.207
Postoperative complications	12 (31.6%)	6 (5.0%)	0.001

### Univariate analysis

Univariate analysis was conducted on all 159 patients to assess the association between potential variables and the development of PTOA. As shown in Table 2, seven variables were found to be significantly associated with PTOA (*p* < 0.05), including comminuted fractures, joint surface impaction, femoral head cartilage injury, non-anatomical fracture reduction, delayed surgery (> 2 weeks), femoral head necrosis, and prolonged surgical duration. Among these factors, non-anatomical reduction quality was associated with a significantly increased risk of PTOA, with patients in the unsatisfactory reduction group showing a 7.9-fold higher risk compared with those who achieved anatomical reduction (95% CI: 3.2–19.4). Patients undergoing surgery more than 2 weeks after injury had a higher incidence of PTOA (65.8%) than those treated within 2 weeks (29.2%).

**Table 2:** Univariate analysis of research factors.

Variable	PTOA Group (*n* = 38)	Non-PTOA group (*n* = 121)	Statistic (t/χ²)	OR (95% CI)	*P* value
Demographic and Clinical Characteristics					
Gender (Male)	24 (63.2%)	73 (60.3%)	χ² = 0.29	0.73 (0.45–1.19)	0.284
Age (years)	47.2 ± 9.8	45.6 ± 10.2	t = 1.01	–	0.317
BMI (kg/m²)	25.2 ± 3.0	24.6 ± 3.1	t = 0.82	–	0.412
Smoking	10 (26.3%)	35 (28.9%)	χ² = 0.23	1.22 (0.65–2.32)	0.632
Hypertension	14 (36.8%)	31 (25.6%)	χ² = 2.78	1.45 (0.94–2.23)	0.095
Diabetes	9 (23.7%)	18 (14.9%)	χ² = 2.09	1.61 (0.85–3.07)	0.148
Osteoporosis	21 (55.3%)	27 (22.5%)	χ² = 5.93	1.54 (0.86–2.74)	0.145
Fracture-Specific Variables					
Comminution	32 (84.2%)	41 (34.2%)	χ² = 5.61	1.84 (1.02–3.31)	0.042
Joint surface impaction	23 (60.5%)	26 (21.7%)	χ² = 5.89	2.12 (1.15–3.90)	0.015
Femoral head cartilage injury	30 (78.9%)	44 (36.7%)	χ² = 3.12	1.73 (0.94–3.18)	0.078
Fracture reduction quality	–	–	χ² = 8.61	–	0.033
Anatomical	12 (31.6%)	74 (61.2%)	–	–	–
Imperfect	22 (57.9%)	42 (34.7%)	–	–	–
Poor	4 (10.5%)	5 (4.1%)	–	–	–
Time to surgery > 2 weeks	25 (65.8%)	35 (29.2%)	χ² = 5.02	2.01 (1.09–3.71)	0.025
Posterior wall defect	–	–	χ² = 7.89	–	0.019
< 33%	8 (21.1%)	42 (34.7%)	–	–	–
33–50%	12 (31.6%)	52 (43.0%)	–	–	–
> 50%	18 (47.4%)	27 (22.3%)	–	–	–
Femoral head necrosis	14 (36.8%)	9 (7.5%)	χ² = 8.93	2.67 (1.39–5.12)	0.003
Fixation type	–	–	χ² = 4.33	–	0.038
Locking plate	26 (68.4%)	84 (69.4%)	–	–	–
Ordinary plate	12 (31.6%)	37 (30.6%)	–	–	–
Injury Mechanism & Associated Injuries					
Mechanism of injury	–	–	χ² = 6.44	–	0.041
Traffic accidents	20 (52.6%)	68 (56.2%)	–	–	–
Fall	12 (31.6%)	33 (27.3%)	–	–	–
Crushing	6 (15.8%)	20 (16.5%)	–	–	–
Associated injuries	–	–	χ² = 8.21	–	0.012
Nerve	8 (21.1%)	12 (9.9%)	–	–	–
Vessel	3 (7.9%)	2 (1.7%)	–	–	–
Surgical and Functional Outcomes					
Intraoperative blood loss (mL)	485.3 ± 100.2	460.1 ± 120.3	t = 2.53	–	0.013
Duration of surgery (min)	156.4 ± 21.2	138.9 ± 18.7	t = 2.91	–	0.004
Injury Severity Score (ISS)	21.7 ± 4.5	19.2 ± 3.6	t = 2.26	–	0.027

### Multivariate analysis

Variables with a p-value < 0.10 in univariate analysis were further included in the multivariate logistic regression model to identify independent predictors of PTOA (Table 3). The regression model demonstrated good fit (Hosmer–Lemeshow test, *p* = 0.832) and discrimination (AUC = 0.891). Seven variables were independently associated with PTOA: comminution (OR = 1.72, *p* = 0.032), joint surface impaction (OR = 1.96, *p* = 0.015), femoral head necrosis (OR = 2.45, *p* = 0.007), non-anatomical reduction (OR = 1.89, *p* = 0.041), posterior wall defect ≥ 50% (OR = 2.11, *p* = 0.014), and time to surgery > 2 weeks (OR = 1.95, *p* = 0.028). Additionally, prolonged surgical duration was associated with increased PTOA risk (OR = 1.14 per 10-minute increment, *p* = 0.004).

**Fig. 2:**
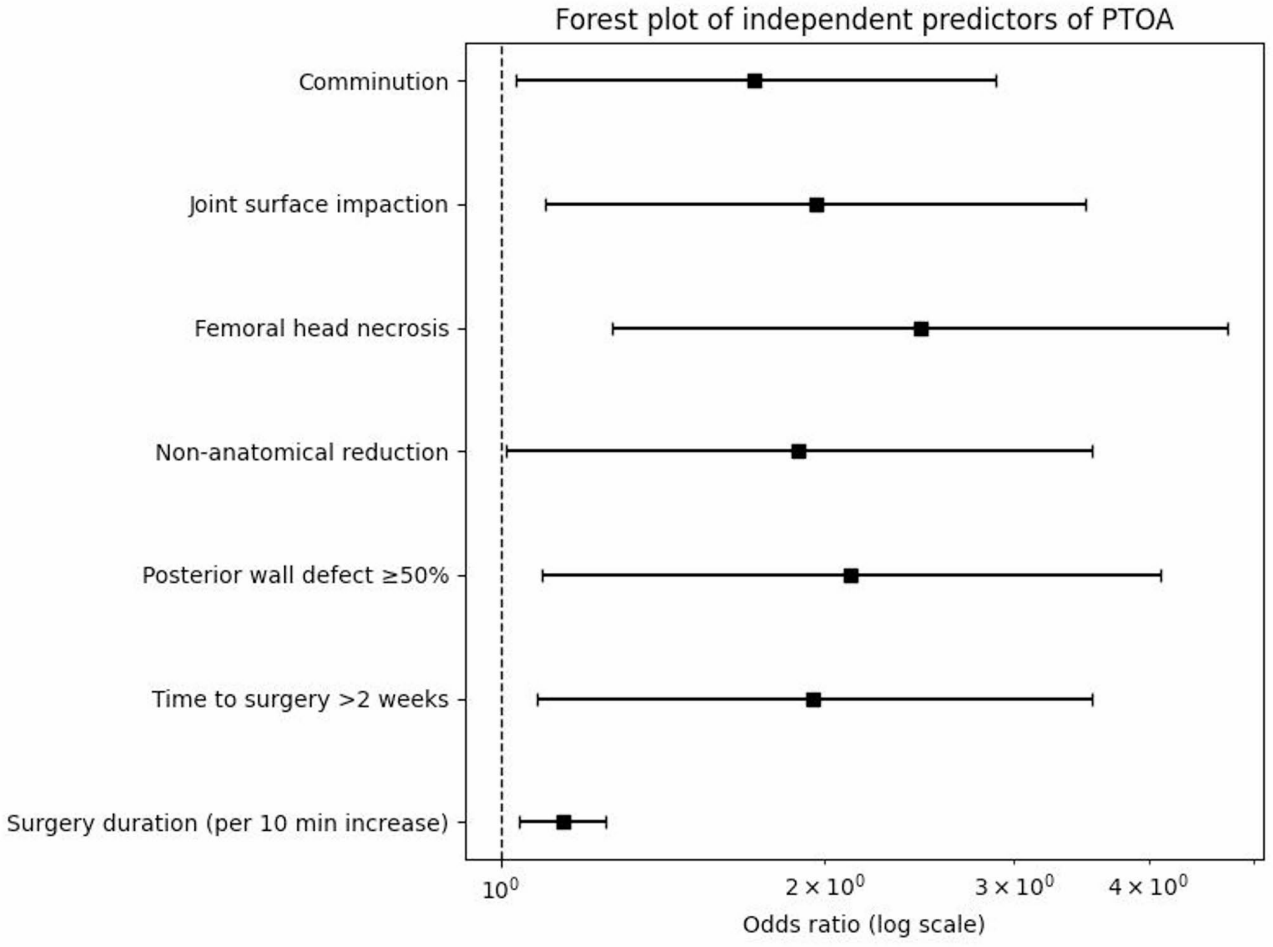
Forest plot of independent predictors of PTOA. The forest plot shows odds ratios (ORs) with 95% confidence intervals (CIs) derived from multivariate logistic regression analysis. Femoral head necrosis, posterior wall defect ≥ 50%, joint surface impaction, non-anatomical reduction, time to surgery > 2 weeks, comminution, and longer surgical duration (per 10-minute increase) were independently associated with an increased risk of PTOA. The vertical dashed line indicates an OR of 1.0.

**Fig. 3:**
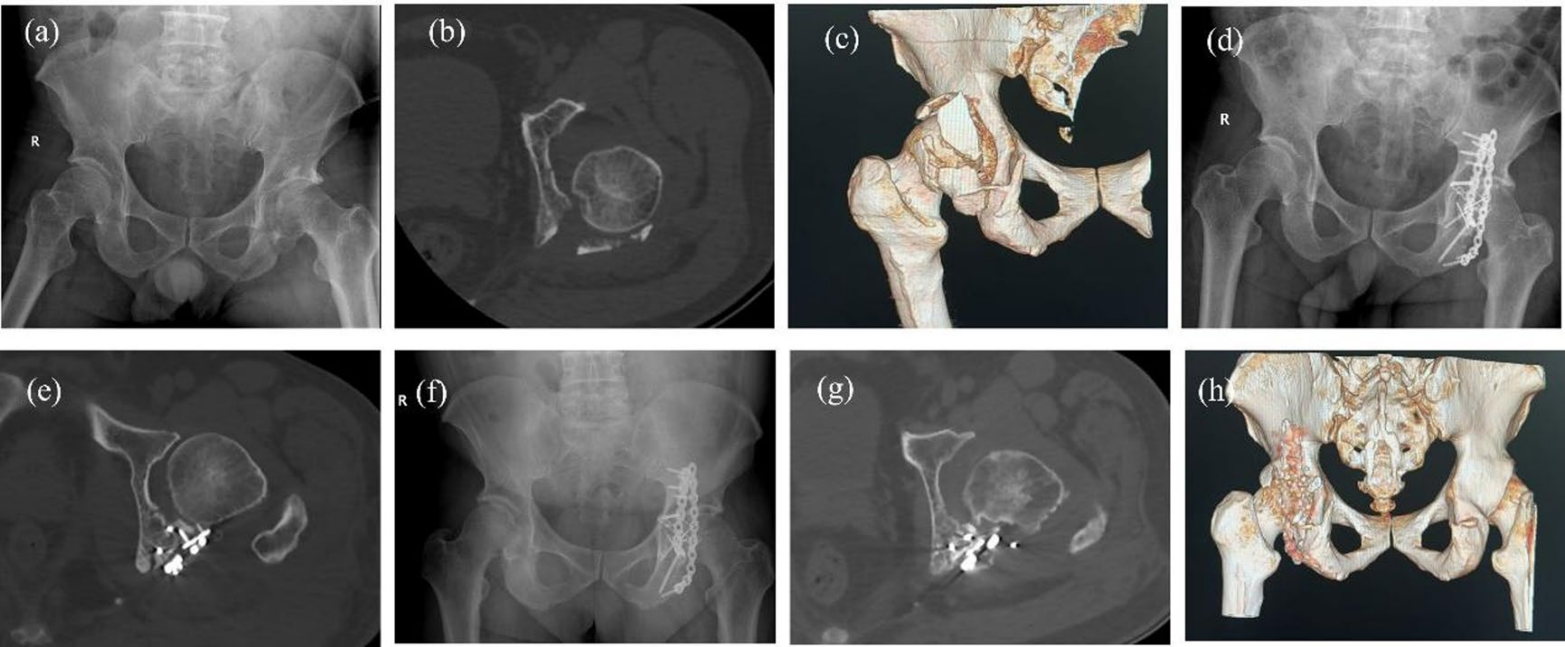
A representative case of PTOA following posterior wall acetabular fracture(**a**-**h**). (**a**) A 55-year-old man sustained a traffic accident resulting in a left posterior wall acetabular fracture. (**b**, **c**) Preoperative CT cross-section and three-dimensional (3D) CT examination are shown. (**d**) The patient underwent posterior wall acetabular fixation with a locking plate. (**e**) Postoperative CT cross-section revealed partial joint surface compression changes. (**f**) One month post-surgery, no significant joint degeneration was observed. (**g**, **h**) Seven months post-surgery, CT cross-section and 3D CT examination showed joint surface sclerosis and surrounding osteophyte formation, indicating the development of PTOA.

The forest plot (Fig. 2) presents the odds ratios (ORs) and 95% confidence intervals (CIs) of independent predictors associated with PTOA. Femoral head necrosis was associated with an increased risk of PTOA (OR = 2.45, 95% CI: 1.27–4.73). A posterior wall defect ≥ 50% (OR = 2.11,95% CI: 1.09–4.10) and joint surface impaction (OR = 1.96, 95% CI:1.10–3.49) were also associated with PTOA. Non-anatomical reduction quality (OR = 1.89, 95% CI: 1.01–3.54), and time to surgery > 2 weeks (OR = 1.95, 95% CI: 1.08–3.54) showed increased odds of PTOA. For regression analyses, reduction quality was dichotomized. Non-anatomical reduction was defined as the presence of either imperfect or poor reduction, while anatomical reduction was used as the reference category. In addition, comminution (OR = 1.72, 95% CI:1.03–2.88) and longer surgery duration (per 10-minute increase, OR = 1.14, 95% CI:1.04–1.25) were associated with higher PTOA risk. Typical cases of PTOA are shown in Fig. 3.

**Table 3:** Multivariate logistic regression analysis of independent risk factors for PTOA.

Variable	B	S.E.	Wald	OR	95% CI	*P* value
Comminution	0.542	0.253	4.58	1.72	1.03–2.88	0.032
Joint surface impaction	0.673	0.276	5.95	1.96	1.10–3.49	0.015
Femoral head necrosis	0.896	0.331	7.31	2.45	1.27–4.73	0.007
Non-anatomical reduction	0.637	0.312	4.17	1.89	1.01–3.54	0.041
Posterior wall defect ≥ 50%	0.747	0.304	6.02	2.11	1.09–4.10	0.014
Time to surgery > 2 weeks	0.668	0.304	4.84	1.95	1.08–3.54	0.028
Surgery duration (per 10 min increase)	0.131	0.046	8.12	1.14	1.04–1.25	0.004

## Discussion

Posterior wall acetabular fractures typically result from high-energy trauma and present significant management challenges due to their frequent association with hip dislocation and femoral head injury. Prevention of complications—particularly post-traumatic osteoarthritis (PTOA)—remains a primary treatment objective. In our cohort, the incidence of PTOA was 23.9%, which aligns with previously reported rates ranging from 19% to 28%^19,20^. This analysis identified several factors associated with the development of PTOA, including fracture comminution, articular surface impaction, non-anatomical reduction quality, femoral head necrosis, and osteoporosis. These factors appear to be closely related to postoperative joint function and long-term clinical outcomes.

Beyond their statistical significance, these factors provide clinically relevant context for risk stratification and individualized surgical planning in patients with complex posterior wall acetabular fractures, where treatment decisions directly influence long-term joint preservation.

The selection of functional thresholds following acetabular fracture surgery remains controversial. While an HHS < 70 is traditionally considered indicative of a poor outcome, higher scores may still reflect clinically meaningful limitations in patients sustaining high-energy trauma. In the present study, the HHS was therefore interpreted as a functional outcome measure rather than a determinant of PTOA development, and was excluded from multivariable analyses to avoid conceptual overlap.

### Fracture comminution and articular surface impaction

Fracture comminution was significantly associated with an increased risk of PTOA, consistent with the findings of Giannoudis et al.^19^, who highlighted the relationship between fracture complexity and postoperative complications. Highly comminuted fractures are often associated with articular incongruity and cartilage damage, which may increase the risk of subsequent joint degeneration. Articular surface impaction was another strong predictor of PTOA in our study. Such impaction leads to subchondral bone injury and promotes early cartilage degeneration^21^. Schenker and Zhao et al. demonstrated that impaction of the joint surface exacerbates chondral injury and triggers intra-articular inflammation, thereby accelerating degenerative changes^22,23^. In addition, a large posterior wall defect (≥ 50%) was associated with an increased risk of PTOA. Extensive posterior wall involvement compromises acetabular containment and joint stability, while concomitant articular surface impaction further exacerbates cartilage damage and disrupts normal joint congruity, collectively predisposing the hip to progressive degenerative changes.

From a clinical standpoint, the coexistence of comminution and articular impaction highlights fracture patterns in which achieving durable joint preservation is particularly challenging, underscoring the need for careful preoperative assessment and realistic discussions regarding long-term joint prognosis.

### Reduction quality and operative time

Reduction quality was a key predictor of PTOA. Suboptimal reduction was associated with a higher incidence of PTOA, consistent with Tannast’s findings^16^ that accurate reduction is vital for joint preservation. Residual step-offs or gaps lead to joint incongruity and abnormal cartilage loading, accelerating degenerative changes^24^. Prolonged operative time was also independently associated with PTOA, likely reflecting greater fracture complexity, increased soft tissue manipulation, and cumulative cartilage insult rather than surgical inefficiency alone^25^. It should be noted that prolonged operative time is unlikely to represent an isolated risk factor and may instead serve as a surrogate marker for fracture complexity and reduction difficulty. Therefore, operative time should be interpreted as a marker of cumulative intraoperative burden rather than an independent causal determinant of PTOA. Taken together, fracture comminution and prolonged operative duration may represent indicators of injury severity and technical complexity, rather than isolated procedural shortcomings. Their association with PTOA further highlights the cumulative impact of mechanical disruption and intraoperative burden on long-term joint preservation.

Importantly, our findings suggest that operative duration should be interpreted in conjunction with reduction quality rather than viewed as an isolated technical target. From an intraoperative decision-making perspective, these results support a balanced approach in which additional surgical time is justified only when it meaningfully improves reduction accuracy, particularly in complex or comminuted fracture patterns. Conversely, prolonged operative time without corresponding gains in reduction quality may increase biological insult without conferring proportional protective benefit against PTOA.

### Timing of surgical intervention

Our findings underscore the importance of timely surgical management. Patients who underwent surgery more than two weeks after injury had a significantly higher risk of PTOA. This observation aligns with prior studies demonstrating that delayed intervention contributes to irreversible chondral damage, compromised femoral head perfusion, and increased difficulty in achieving anatomical reduction, all of which adversely affect long-term joint function^26,27^. Early intervention improves the technical feasibility of reduction and reduces the likelihood of long-term complications^28^. Clinically, these findings emphasize the importance of early referral and timely decision-making, as treatment delays may limit reconstructive potential and should be considered during preoperative counseling regarding expected outcomes and long-term joint health.

### Femoral head necrosis and osteoporosis

Femoral head necrosis emerged as an independent predictor of PTOA. Notably, femoral head necrosis demonstrated the largest effect size among the identified predictors in our analysis. This finding underscores the critical role of femoral head viability in maintaining joint integrity, as ischemic damage may precipitate structural collapse, altered load transmission, and rapid degeneration of the articular surface. As Zhao et al. pointed out, avascular necrosis of the femoral head—due to disrupted blood supply—can result in structural collapse and accelerate joint surface degeneration^23^. A meta-analysis further demonstrated that patients with femoral head impaction have an approximately sixfold increased risk of developing PTOA^29^.

Osteoporosis was evaluated preoperatively using dual-energy X-ray absorptiometry; however, it did not show a statistically significant association with PTOA in univariate analysis and was therefore not included as an independent predictor in the multivariate model. Nevertheless, bone quality remains a clinically relevant consideration in the management of acetabular fractures.

Decreased bone mineral density may compromise screw purchase and subchondral support, potentially increasing micromotion at the fracture site and affecting long-term joint stability. Although osteoporosis did not reach statistical significance in our cohort, previous studies have suggested that poor bone quality may predispose patients to fixation-related complications and inferior outcomes following acetabular fracture surgery. Khalifa et al. reported higher rates of nonunion and joint instability in osteoporotic patients^30^, and Arabnejad et al. demonstrated through finite element modeling that osteoporosis substantially reduces the mechanical strength of the acetabular region, increasing the risk of implant loosening and fixation failure^31^. Taken together, these findings suggest that while osteoporosis was not an independent risk factor for PTOA in the present study, it may still influence surgical strategy and postoperative management, particularly in patients with complex fractures or compromised fixation conditions.

### Fracture severity and postoperative complications

Our results reaffirm the role of fracture comminution and articular impaction as strong predictors of PTOA. joint surface damage and the extent of comminution are key determinants of both prognosis and postoperative complication rates in complex acetabular fractures^20^. Articular impaction triggers a cascade of inflammatory responses in damaged cartilage, accelerating joint degeneration^29^. These mechanisms support our findings on the critical role of joint surface morphology in PTOA development.

### Importance of early reduction and stable fixation

Our study highlights the importance of early surgery and accurate reduction in reducing PTOA risk. Miller et al. demonstrated that early intervention lowers joint instability and cartilage injury, thereby improving outcomes^32^. Locking plates offer improved fixation stability compared with conventional plates and facilitate anatomical reduction, particularly in comminuted or osteoporotic fractures^33^. Biomechanical studies have shown that locking constructs better withstand cyclic loading, promoting fracture healing and reducing complications such as nonunion and PTOA^34,35^. In our cohort, higher PTOA rates were observed with conventional plate fixation, suggesting that fixation stability may influence long-term joint outcomes, especially in fractures with compromised bone quality or severe comminution. These findings suggest that suboptimal biomechanical support may contribute to PTOA development. Delayed surgical intervention, inadequate reduction quality, and the use of conventional non-locking plates may fail to restore sufficient joint stability and load distribution, thereby increasing cartilage stress and accelerating degenerative changes, particularly in fractures with severe comminution or compromised bone quality.

It should be noted that fixation strategy in this study was not randomized and was influenced by multiple clinical factors. Locking plate constructs were generally selected for fractures with greater comminution, compromised bone quality, or larger posterior wall defects, where enhanced angular stability was considered advantageous. In contrast, conventional plates were more often used in patients with relatively preserved bone stock and less complex fracture patterns. Surgeon preference and institutional practice patterns also contributed to implant selection. As a result, the observed association between fixation type and PTOA may be subject to confounding by fracture severity and bone quality, and should be interpreted with caution rather than as a causal relationship.

Additionally, lower postoperative HHS were associated with the development of PTOA, reflecting pain, stiffness, and functional limitation. In our cohort, non-anatomical reduction quality and femoral head necrosis were frequently accompanied by lower Harris scores, indicating that mechanical and biological injury may synergistically contribute to functional decline. This finding reinforces the close relationship between surgical reconstruction quality, biological integrity, and patient-reported functional outcomes. Although lower postoperative HHS were observed in patients who developed post-traumatic osteoarthritis, functional impairment after acetabular fracture is multifactorial. Factors such as residual soft-tissue injury, muscle weakness, pain, rehabilitation adherence, and concomitant injuries may substantially influence postoperative functional recovery and are not solely attributable to degenerative joint changes. Importantly, an HHS threshold of 70 is not generally considered indicative of a poor outcome, and functional scores should therefore be regarded as downstream clinical manifestations rather than definitive markers of osteoarthritis.

### Long-term functional impact and role of arthroplasty

Post-traumatic osteoarthritis has a profound impact on long-term function and is a leading cause of late-stage conversion to total hip arthroplasty (THA)^36^. Radiographic progression of PTOA may occur despite initially satisfactory recovery, underscoring the insidious nature of cartilage degeneration^6,8,37^. Approximately 30% of patients with initially preserved hip joints were reported to require THA within 10 years following internal fixation^7,17^.With advancements in arthroplasty techniques, some studies have proposed primary THA as an alternative in select patients with complex fractures—particularly in the elderly^38,39^. However, further prospective studies with longer follow-up, standardized imaging protocols, and advanced cartilage assessment (e.g., MRI T2 mapping) are warranted to validate these findings and further elucidate the biomechanical pathways leading to PTOA.

Several limitations of this study should be acknowledged. First, the retrospective design is susceptible to selection bias and unmeasured confounding, despite adjustment for known risk factors using multivariable analysis. Second, although preoperative bone mineral density was assessed using DEXA and osteoporosis was defined based on established criteria, preoperative osteoarthritis (OA) patterns were not systematically evaluated, precluding analysis of the potential influence of OA morphology on surgical and clinical outcomes.

In addition, postoperative functional outcomes assessed by the HHS are influenced by multiple factors during fracture healing, including pain, muscle strength, rehabilitation adherence, and soft-tissue recovery, and should therefore be interpreted as associated clinical manifestations rather than direct surrogates for PTOA progression. Residual confounding cannot be entirely excluded given the limited sample size relative to the number of potential predictors. Furthermore, variability in surgical technique, surgeon experience, and intraoperative decision-making across centers may have influenced outcomes but was not fully captured. Finally, the generalizability of these findings may be limited, and prospective multicenter studies are warranted.

## Conclusion

This study identified multiple independent factors associated with PTOA, including fracture comminution, joint surface impaction, non-anatomical reduction quality, femoral head necrosis, posterior wall defect, and delayed surgical intervention. Among these, anatomical reduction and earlier surgical intervention were associated with a lower risk of PTOA in our cohort. These findings underscore the potential importance of early and precise surgical management in preserving hip joint function.

## Data Availability

The datasets used or analyzed during the current study are available from the corresponding author on reasonable request.

## Abbreviations

PTOA: Post-traumatic osteoarthritis
ORIF: open reduction and internal fixation
CT: Computed tomography
3D: Three-dimensional
HHS: Harris Hip Score
THA: Total hip replacement
BMI: Body mass index

## References

[1] Albrektsson, M., Möller, M., Wolf, O., Wennergren, D. & Sundfeldt, M. Acetabular fractures: Epidemiology and mortality based on 2,132 fractures from the Swedish Fracture Register. *Bone Jt. Open.* **4**, 652–658. 10.1302/2633-1462.49.BJO-2023-0085.R1 (2023). 37652452 10.1302/2633-1462.49.BJO-2023-0085.R1 PMC10471445

[2] McNamara, A. R., Boudreau, J. A. & Moed, B. R. Nonoperative Treatment of Posterior Wall Acetabular Fractures After Dynamic Stress Examination Under Anesthesia: Revisited. *J. Orthop. Trauma.* **36**, S1–6. 10.1097/BOT.0000000000002344 (2022). 35061643 10.1097/BOT.0000000000002344

[3] Perdue, P. W., Tainter, D., Toney, C. & Lee, C. Evaluation and Management of Posterior Wall Acetabulum Fractures. *J. Am. Acad. Orthop. Surg.* **29**, e1057–e1067. 10.5435/JAAOS-D-20-01301 (2021). 34323866 10.5435/JAAOS-D-20-01301

[4] Ziran, N., Soles, G. L. S. & Matta, J. M. Outcomes after surgical treatment of acetabular fractures: a review. *Patient Saf. Surg.* **13**, 16. 10.1186/s13037-019-0196-2 (2019). 30923570 10.1186/s13037-019-0196-2 PMC6420740

[5] Ferguson, T. A., Patel, R., Bhandari, M. & Matta, J. M. Fractures of the acetabulum in patients aged 60 years and older: an epidemiological and radiological study. *J. Bone Joint Surg. Br.* **92**, 250–257. 10.1302/0301-620X.92B2.22488 (2010). 20130318 10.1302/0301-620X.92B2.22488

[6] Li, J. et al. Predictors for post-traumatic hip osteoarthritis in patients with transverse acetabular fractures following open reduction internal fixation: a minimum of 2 years’ follow-up multicenter study. *BMC Musculoskelet. Disord*. **24**, 811. 10.1186/s12891-023-06945-2 (2023). 37833696 10.1186/s12891-023-06945-2 PMC10571302

[7] Magu, N. K. et al. Long term results after surgical management of posterior wall acetabular fractures. *J. Orthopaed Traumatol.* **15**, 173–179. 10.1007/s10195-014-0297-8 (2014). 10.1007/s10195-014-0297-8 PMC4182623 24879360

[8] Rommens, P. M., Ingelfinger, P., Nowak, T. E., Kuhn, S. & Hessmann, M. H. Traumatic damage to the cartilage influences outcome of anatomically reduced acetabular fractures: A medium-term retrospective analysis. *Injury* **42**, 1043–1048. 10.1016/j.injury.2011.03.058 (2011). 21513934 10.1016/j.injury.2011.03.058

[9] Letournel, E. Acetabulum fractures: classification and management. *Clin. Orthop. Relat. Res.* **151**, 81–106 (1980). 7418327

[10] Cosgrove, C. T., Berkes, M. B., McAndrew, C. M. & Miller, A. N. Kocher-Langenbeck Approach for Posterior Wall Acetabular Fractures. *J. Orthop. Trauma.* **34** (Suppl 2), S21–S22. 10.1097/BOT.0000000000001816 (2020). 32639344 10.1097/BOT.0000000000001816

[11] Matta, J. M. Fractures of the acetabulum: accuracy of reduction and clinical results in patients managed operatively within three weeks after the injury. *J. Bone Joint Surg. Am.* **78**, 1632–1645 (1996). 8934477

[12] Hsu, C-L. et al. A Simple Method for Calculating Acetabular Posterior Wall Fracture Fragment Percentages on Three-Dimensional Computed Tomography Scan Reconstruction Images. *J. Med. Sci.* **43**, 269–275. 10.4103/jmedsci.jmedsci_244_22 (2023).

[13] Ficat, R. P. Idiopathic bone necrosis of the femoral head. Early diagnosis and treatment. *J. Bone Joint Surg. Br.* **67**, 3–9. 10.1302/0301-620X.67B1.3155745 (1985). 3155745 10.1302/0301-620X.67B1.3155745

[14] Brooker, A. F., Bowerman, J. W., Robinson, R. A. & Riley, L. H. Ectopic ossification following total hip replacement. Incidence and a method of classification. *J. Bone Joint Surg. Am.* **55**, 1629–1632 (1973). 4217797

[15] AH Eren. Harris hip score. *Acta Orthop. Traumatol. Turc.* **31**, 285–288 (1997).

[16] Tannast, M., Najibi, S. & Matta, J. M. Two to twenty-year survivorship of the hip in 810 patients with operatively treated acetabular fractures. *J. Bone Joint Surg. Am.* **94**, 1559–1567. 10.2106/JBJS.K.00444 (2012). 22992846 10.2106/JBJS.K.00444

[17] Verbeek, D. O., Van Der List, J. P., Tissue, C. M. & Helfet, D. L. Long-term patient reported outcomes following acetabular fracture fixation. *Injury* **49**, 1131–1136. 10.1016/j.injury.2018.04.031 (2018). 29729818 10.1016/j.injury.2018.04.031

[18] Pai, V., Bell, D. & Knipe, H. Kellgren and Lawrence system for classification of osteoarthritis. Radiopaedia.org [Internet]. Radiopaedia.org; [cited 2025 July 8]. 10.53347/rID-27111 (2014).

[19] Giannoudis, P. V., Grotz, M. R. W., Papakostidis, C. & Dinopoulos, H. Operative treatment of displaced fractures of the acetabulum. A meta-analysis. *J. Bone Joint Surg. Br.* **87**, 2–9 (2005). 15686228

[20] Dwight, K. D. & Maceroli, M. Posttraumatic Arthritis After Acetabular Fractures. *Orthop. Clin. North Am.* **55**, 453–459. 10.1016/j.ocl.2024.04.007 (2024). 39216950 10.1016/j.ocl.2024.04.007

[21] Ochi, H. et al. Acetabular cartilage abnormalities in elderly patients with femoral neck fractures. *SICOT J.* **8**, 24. 10.1051/sicotj/2022022 (2022). 35699460 10.1051/sicotj/2022022 PMC9196023

[22] Schenker, M. L., Mauck, R. L., Ahn, J. & Mehta, S. Pathogenesis and Prevention of Posttraumatic Osteoarthritis After Intra-articular Fracture. *J. Am. Acad. Orthop. Surg.* **22**, 20–28. 10.5435/JAAOS-22-01-20 (2014). 24382876 10.5435/JAAOS-22-01-20 PMC4425936

[23] Zhao, D. et al. Guidelines for clinical diagnosis and treatment of osteonecrosis of the femoral head in adults (2019 version). *J. Orthop. Translation*. **21**, 100–110. 10.1016/j.jot.2019.12.004 (2020). 10.1016/j.jot.2019.12.004 PMC7152793 32309135

[24] Cimerman, M., Kristan, A., Jug, M. & Tomaževič, M. Fractures of the acetabulum: from yesterday to tomorrow. *Int. Orthop. (SICOT)*. **45**, 1057–1064. 10.1007/s00264-020-04806-4 (2021). 10.1007/s00264-020-04806-4 PMC8052228 32964295

[25] Ibaseta, A. et al. Effect of operative time in outcomes following surgical fixation of hip fractures: a multivariable regression analysis of 35,710 patients. *Hip Int.* **34**, 270–280. 10.1177/11207000231203527 (2024). 37795582 10.1177/11207000231203527

[26] Muzzammil, M. et al. Timing of surgical intervention for acetabular fractures: A literature review of outcomes and experiences in low resource settings. *J. Orthop. Rep.* **2**, 100191. 10.1016/j.jorep.2023.100191 (2023).

[27] Enocson, A. & Lundin, N. Early versus late surgical treatment of pelvic and acetabular fractures a five-year follow-up of 419 patients. *BMC Musculoskelet. Disord*. **24**, 848. 10.1186/s12891-023-06977-8 (2023). 37891518 10.1186/s12891-023-06977-8 PMC10605968

[28] Wójcicki, R. et al. The Association between Acetabulum Fractures and Subsequent Coxarthrosis in a Cohort of 77 Patients—A Retrospective Analysis of Predictors for Secondary Hip Osteoarthritis. *JCM* **12**, 6553. 10.3390/jcm12206553 (2023). 37892691 10.3390/jcm12206553 PMC10607311

[29] Esmaeili, S. et al. Risk factors for acetabular fracture treatment failure: a systematic review and meta-analysis. *BMC Musculoskelet. Disord*. **25**, 976. 10.1186/s12891-024-08114-5 (2024). 39609839 10.1186/s12891-024-08114-5 PMC11605918

[30] Khalifa, A. A., Mahran, D. G., Fergany, A. & Farouk, O. Epidemiology of acetabular fractures in elderly patients and the effect of various management options on the outcomes. A comprehensive narrative review. *Int. J. Orthop. Trauma. Nurs.* **53**, 101049. 10.1016/j.ijotn.2023.101049 (2024). 37852917 10.1016/j.ijotn.2023.101049

[31] Khakpour, S. et al. Effect of osteoporosis-related reduction in the mechanical properties of bone on the acetabular fracture during a sideways fall: A parametric finite element approach. Tomaszewska E, editor. *PLoS ONE. Public Library of Science (PLoS)* **17**, e0263458 (2022). 10.1371/journal.pone.0263458 10.1371/journal.pone.0263458 PMC8820641 35130332

[32] Cahueque, M., Martínez, M., Cobar, A. & Bregni, M. Early reduction of acetabular fractures decreases the risk of post-traumatic hip osteoarthritis? *J. Clin. Orthop. Trauma.* **8**, 320–326. 10.1016/j.jcot.2017.01.001 (2017). 29062212 10.1016/j.jcot.2017.01.001 PMC5647687

[33] Shibata, R. et al. Outcomes of spring-locking plate fixation method using locking mesh plate/box plate for posterior wall fractures of the acetabulum: a retrospective single-centre study. *Injury* **55**, 111172. 10.1016/j.injury.2023.111172 (2024). 37951016 10.1016/j.injury.2023.111172

[34] Mehin, R., Jones, B., Zhu, Q. & Broekhuyse, H. A biomechanical study of conventional acetabular internal fracture fixation versus locking plate fixation. *Can. J. Surg.* **52**, 221–228 (2009). 19503667 PMC2689727

[35] Lodde, M. F. et al. Angular stable plate fixation provides favorable biomechanical stability in simulated T-shaped acetabular fractures: a biomechanical study. *Acta Orthop.* **95**, 701–706. 10.2340/17453674.2024.42490 (2024). 39607368 10.2340/17453674.2024.42490 PMC11603667

[36] Chen, H. Y. & Tsai, Y. H. Risk factors for post-traumatic osteoarthritis and subsequent total hip arthroplasty in patients with acetabular fractures. *BMC Musculoskelet. Disord*. **26**, 440. 10.1186/s12891-025-08690-0 (2025). 40325376 10.1186/s12891-025-08690-0 PMC12051267

[37] Tucker, A. et al. Evaluation of the trajectory of recovery following surgically treated acetabular fractures. *Bone Joint J.* **106-B**, 69–76. 10.1302/0301-620X.106B1.BJJ-2023-0499.R2 (2024). 38160696 10.1302/0301-620X.106B1.BJJ-2023-0499.R2

[38] Enocson, A. & Chang, D. Acetabular fractures in the elderly treated with acute fixation and primary total hip arthroplasty: a 3-year follow-up of 70 patients. *Arch. Orthop. Trauma. Surg.* **145**, 323. 10.1007/s00402-025-05941-6 (2025). 40445248 10.1007/s00402-025-05941-6 PMC12125119

[39] Smakaj, A. et al. Mid-term outcomes of acetabular fractures treated with acute fix and replace versus ORIF in the elderly: a multicentric study with minimum 5-year follow-up. *Eur. J. Orthop. Surg. Traumatol.* **35**, 192. 10.1007/s00590-025-04309-1 (2025). 40366425 10.1007/s00590-025-04309-1 PMC12078442

